# Understanding access to sexual and reproductive health in general practice using an adapted Candidacy Framework: a systematic review and qualitative evidence synthesis

**DOI:** 10.3399/BJGP.2024.0522

**Published:** 2025-06-17

**Authors:** Rebecca L Mawson, Victoria Hodges, Sarah Salway, Caroline Mitchell

**Affiliations:** 1 School of Medicine and Population Health, University of Sheffield, Sheffield, UK; 2 Primary Care School, Keele University, Keele, UK; 3 Department of Sociological Studies, University of Sheffield, Sheffield, UK

**Keywords:** primary health care, delivery of health care, sexually transmitted infections, qualitative research, socioeconomic factors, systematic review

## Abstract

**Background:**

General practice has a key role in reducing inequity in access to care relating to sexual and reproductive health (SRH). Unplanned pregnancy, abortion, and sexually transmitted infections are increasing and disproportionately affect deprived communities and minoritised ethnic groups. The Candidacy Framework is a practical and theoretical framework for understanding the complex interactional processes of access to SRH care in general practice.

**Aim:**

To use the Candidacy Framework to explore access to SRH care in general practice. The seven interaction stages are: identification of need; navigation of services; permeability of services; appearing and asserting need; adjudication by healthcare professional (HCP); offers or resistance of offer; and the local operating conditions or local production of candidacy.

**Design & setting:**

Systematic review with qualitative evidence synthesis using a framework approach.

**Method:**

A systematic search of MEDLINE, Embase, PubMed, and the Web of Science was conducted to identify primary qualitative research exploring access to SRH care in general practice from practitioner, public, and patient perspectives in countries with universal health care. The Candidacy Framework was used to synthesise the findings.

**Results:**

Analysis of 42 studies revealed the impact of stigma, shame, and embarrassment among individuals, communities, and HCPs. Findings showed limited inclusion of demographics, such as ethnicity and socioeconomic status. Barriers to access were more evident for those from lower socioeconomic communities, minoritised ethnic groups, and the LGBTQ+ community. There are multiple barriers, which include the behaviours of HCPs, who have a crucial role in recognising an individual’s SRH need.

**Conclusion:**

General practice offers a cradle-to-grave healthcare service that should have SRH as a priority area of provision. Further understanding is needed about the impact of historic harms by medicine and health care on racialised individuals and minoritised genders.

## How this fits in

Healthcare practitioners (HCPs) can obstruct access to health care. Understanding HCPs’ beliefs affecting access is key to improving recognition and acceptance of candidacy. Better comprehension of complex decision-making and personal choice is needed for policy planning. Stigma, shame, misinformation, and fear persist in society and medicine regarding sexual and reproductive health (SRH). The historic legacy of harm within SRH services must be understood to repair structural healthcare barriers. SRH access in primary care is complex, rooting in education, empowerment, and communication. While primary care provides most NHS contraceptive care, LGBTQ+ individuals and people with HIV often feel excluded. Research must include participants’ ethnicity and socioeconomic status. Underserved communities are being failed by this lack of visibility in research.

## Introduction

Sexual and reproductive health (SRH) affects everyone at some point in their life, whether that be in terms of family planning, contraception, safe sex, or relationships. The UK has seen a reduction in the funding of SRH services in the context of increasing rates of sexually transmitted infections (STIs), as well as high unplanned pregnancy and abortion rates.^1,2^ In the broader context of Europe, SRH care is provided by various providers, with responsibility falling to different specialities;^3^ this is predominantly in general practice in the UK and the Netherlands, with Sweden and Portugal providing community health centres. Gynaecologists working at a primary care level offer services for women in Germany and Poland; primary care providers do so for men. In the UK, 59.1% of contraception and 17.2% of chlamydia screening is provided in general practice, yet services are variable and inconsistent between surgeries.^4,5^

The COVID-19 pandemic has led to further challenges in access to SRH care, as seen in other areas of health care,^6,7^ and NHS Digital^8^ data have shown a fall in long-acting reversible contraception (LARC) prescriptions (such as intrauterine devices, implants, and injections) in GP surgeries since COVID-19 hit. In 2021, there were 214 256 abortions for women in England and Wales, the highest number since the Abortion Act was established in 1967.^5^ Abortion rates in the UK are higher compared with other high-income countries; a recent report by the British Pregnancy Advisory Service^9^ found key factors to include the cost-of-living crisis, accessibility of abortion, and contraception acceptability. The highest burden of poor access and adverse outcomes falls on patient groups that are marginalised by sexual orientation, gender/transgender status, minoritised ethnicity, socioeconomic deprivation, and, especially, young age (adolescence).^10–13^ Similar patterns are seen across Europe and North America, with worse outcomes for underserved populations, such as migrant women, Black and minoritised ethnic groups, and LGBTQ+ communities.^14–16^

There are a range of conceptualisations of healthcare access, and some focus on the supply of services meeting the demand of service users; when supply equals demand, access is no longer an issue.^17,18^ Levesque *et al* ^19^ developed a holistic conceptual framework based on broader definitions of access to, and accessibility of, healthcare services. Unfortunately, using narrow interpretations of access can lead to lack of clarity about various services and the groups seeking to access them. Choice can easily be simplified into the concept of an individual decision. Policies driven at improving the range of choice — of, for example, contraception — may not improve uptake because far wider influences are at play than availability.^20^

Dixon-Woods *et al* ^21^ developed the concept of ‘candidacy’ in 2005, from a literature synthesis focused on studies exploring inequalities in healthcare access and utilisation. The dynamic and inter-relational stages help to show granularity of access to primary care, which has become a contentious political subject.^22^ Candidacy offers a framework to conceptualise help seeking, healthcare structure, and access, as described in Box 1.^23,24^ The candidacy model has been used previously to explore a range of topics, including colorectal cancer,^25^ asthma management in a South Asian community in Britain,^26^ domestic violence,^27^ and medical homelessness for women in prison.^28^ To the authors’ knowledge, it has not been used to examine SRH care in primary care. A limitation of the candidacy model is that it neglects the influence of wider contexts in which health care is accessed, the historic shaping of medicine that impacts trust, and willingness to seek care. For racialised groups and gender minorities, these intersectional factors lead to worsening disparities and inequity of access.^29^

Access to SRH care in general practice is vital to ensuring equitable and accessible provision of SRH care for all. There has been extensive publication of barriers and facilitators to the provision of SRH care, with barriers including communication challenges, stigma, personal belief systems, and lack of time, knowledge, and/or equipment.^30–32^ Studies focus on specific topics — such as, abortion, sexuality, or communication — and there is a lack of research on access to SRH services in primary care, using an umbrella lens of all areas of SRH care that fall within this context. Using a framework approach with the highly salient candidacy model allows overlapping pathologies and often challenging contexts to be clarified.

## Method

A systematic evidence synthesis using a ‘best-fit’ framework, as described by Carrol *et al*,^33^ was selected for this research. Framework synthesis was chosen over other forms of synthesis because of the complexity and heterogeneity of the data, which required a structural analysis.^34^ A scoping review was performed to find models or theories of access to health care. The Candidacy Framework was identified as the *a priori* framework for the synthesis; an example of how the seven interaction stages could relate to SRH care is summarised in Table 1. Cochrane guidance^35^ was used for the qualitative evidence synthesis and followed the Preferred Reporting Items for Systematic Review and Meta-Analyses (PRISMA) checklist.^36^

**Table 1.** The Candidacy Framework described in the context of SRH, adapted from Dixon-Woods *et al* ^21,23^

Candidacy Framework	Definitions adapted from the work of Dixon-Woods *et al* ^21,23^	Examples of SRH access
**Identification of candidacy**	A person’s recognition of, and response to, a symptom. Influenced by own knowledge, health literacy, community behaviour.	Identifying need for an STI screen due to having had unprotected sex.
**Navigation of services**	A person’s awareness of what services are available and ability to mobilise the practical resources or assets needed to attend the service.	Taking time off work to travel on two buses to get to a central sexual health clinic for an STI screen.
**Permeability of services**	More-porous services require fewer qualifications of candidacy and less mobilisation of resources to attend. Less-permeable services require a higher degree of cultural alignment — for example, navigating booking systems, needing to read appointment letters.	Needing to call the clinic at 8.30 am each morning, navigate a telephone automated system, and speak to a receptionist to book an STI screen.
**Appearing and asserting candidacy**	A person making a claim to candidacy for medical attention or intervention. They need to provide a coherent history and articulate the issue, and have formulated a health need, which requires a level of understanding.	A person asking for condoms in a GP appointment.
**Adjudication**	HCPs judging the worthiness of the candidacy claim and interlinks with perceived social deservingness.	GP not suggesting an implant as a contraception method because the patient has been unreliable at attending appointments in the past.
**Offers of/resistance to services**	An intervention or treatment course has been offered, but declined by the person in need.	Declining offer of cervical screening when attending for a practice nurse health check.
**Operating conditions and local production of candidacy**	Interactions between HCP and patient that can be affected over time — this includes the perceived or actual availability and suitability of resources in a particular setting.	GP turning a patient away when they ask for an HIV test, because it is perceived as not being funded in primary care.

HCP = healthcare professional. SRH = sexual and reproductive health. STI = sexually transmitted infection.

### Selection criteria

The review presented here sought to identify primary qualitative research completed in high-income countries, as defined by the Organisation for Economic Cooperation and Development.^37^ The searches were limited to the English language and full-text articles; case reports, reviews, and conference abstracts were excluded. The publication date was limited to a 25-year period (2000–2024), the rationale being that the National Institute for Health and Care Excellence guidance for LARC^38^ was published in 2005, which likely led to the widespread use of these more modern methods (increased LARC compared to condoms or contraceptive pills). Inclusion and exclusion criteria are given in Supplementary Box S1.

### Search strategy

The MEDLINE, Embase, PubMed, and Web of Science electronic databases were searched, initially in 2018, and then again in July 2024; the date range used was 2000–2024. Results were compiled using Mendeley Reference Manager and imported into the systematic review management software, Covidence (Veritas Health Innovation) (approved by Cochrane). Key domains included ([general practice] OR [practice nurse]), AND ([contraception], OR [sexually transmitted infection]); the full electronic search strategy, including Medical Subject Headings terms, is included in Supplementary Appendix S1.

Box 1. **Candidacy, as defined by Dixon-Woods *et al* ^24^**

*‘Candidacy describes the ways in which people’s eligibility for medical attention and intervention is jointly negotiated between individuals and health services ... Candidacy is a dynamic and contingent process, constantly being defined and redefined through interactions between individuals and professionals ...* [and] *managed in the context of operating conditions ...* [including the biography of the relationship between patients and staff,] *the typifications staff use in categorising people and diseases, availability of economic and other resources such as time, local pressures, and policy imperatives.’*

Two authors independently screened abstracts and conducted full-text reviews, adhering to the inclusion and exclusion criteria. After discussion within the team, certain potential subjects were excluded, not because they were unimportant, but because of the number of publications within the topic. A pragmatic decision was made to exclude these — such as abortion and sexual dysfunction — to make the data manageable. Conflicts were resolved through discussion between the two authors conducting the searches and one other author.

**Figure 1.**
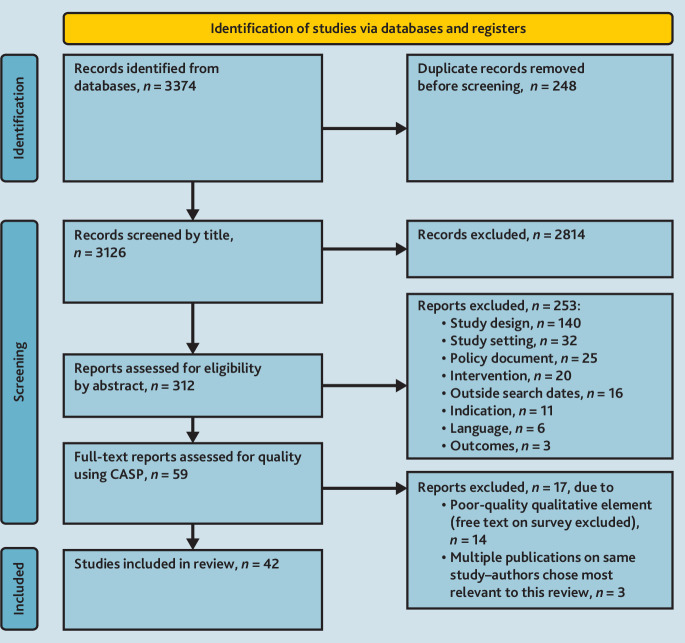
PRISMA 2020 flow diagram of selection process for new systematic reviews that included searches of databases and registers only. CASP = Critical Appraisal Skills Programme. PRISMA = Preferred Reporting Items for Systematic reviews and Meta-Analyses.

Figure 1 shows the PRISMA diagram outlining the selection process.

### Quality summary

Quality assessment of 59 full-text articles was carried out using the Critical Appraisal Skills Programme (CASP) tool for qualitative studies.^39^ Three authors reviewed the full-text articles independently. All included studies were judged to have taken steps to reduce bias; some did not have sufficient details to meet all CASP criteria but, in the discussion as a research team, they were included as they brought rich themes for analysis.^35^

### Charting the data: summary and synthesis

Two approaches were used to chart the data: summary and synthesis. First, the data were extracted from the full-text articles using NVivo.^40^ The background and methodological information was identified using criteria adapted from Cochrane guidance.^35^ Framework analysis was used to explore the data, with themes discussed by all authors; this comprised five stages: familiarisation, identifying a thematic framework (in this case, candidacy), indexing, charting, and mapping and interpreting.^41^ Initially, themes were coded against the existing stages of the Candidacy Framework. Within this synthesis, the viewpoints of healthcare professionals (HCPs) and members of the public were included to draw similarities and differences. The demographic and diversity analysis of participant characteristics was analysed using an Microsoft Excel database.

## Results

In total, 42 qualitative studies were identified;^42–83^ these comprised results from Australia,^^43,44,50,55,63,68,72,77,83^^ Canada,^^71^^ England,^42,45–49,52–54,56,60–62,69,70,74,76,78,80,82^ Ireland,^^57,58^^ Germany,^64,67^ Norway,^59^ the Netherlands,^79^ New Zealand,^65,66^ Scotland,^51,73^ Great Britain,^75^ and the UK.^81^ Participants included HCPs, members of the public, or service users; some studies included views from >1 group. Supplementary Table S1 summarises the included studies.

Twenty studies included participants who were members of the public or patients;^42–49,53–60,62,63,78,83^ this totalled 632 participants, 58% female and 42% male, as identified by the studies. Age ranges for the studies varied considerably — between 15 years^58^ and 92 years.^52^ In terms of diversity, 11 of the 20 non-HCP studies described participant ethnicity or ethnic group.^42,45,46,48,49,53–56,62,78^ One of the 20 studies included a focus group in Punjabi,^42^ which was conducted by a bilingual researcher. Three of the 20 studies^43–45^ specifically excluded participants who were not proficient in English. Nine studies^45–53^ mentioned that purposive sampling had been used to increase diversity of ethnicity/ethnic group and/or deprivation status. Education level or occupation was sometimes used as a proxy representation of social status. Four studies^43,53–55^ included occupation, employment status, or income of the participant, and three studies^42,53,56^ included educational background. Study characteristics are presented in Supplementary Table S1.

**Figure 2.**
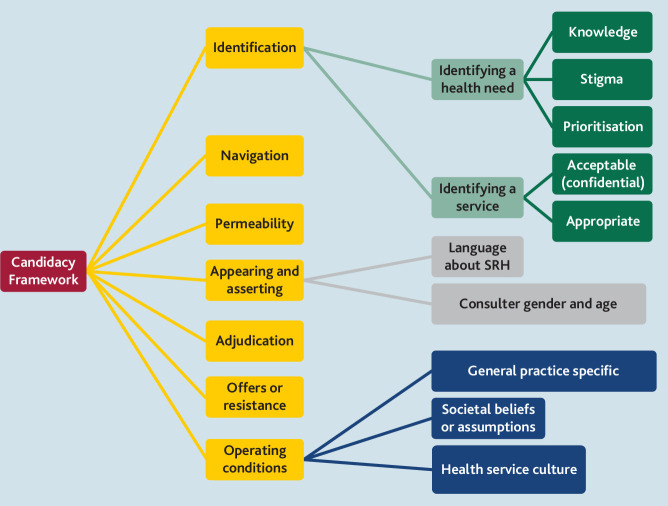
Summary of candidacy themes developed in the synthesis. SRH = sexual and reproductive health.

The results are presented using the seven stages of the Candidacy Framework. These are not distinct stages, and have overlapping and interacting relationships. A visual representation of the analysis can be seen in Figure 2.

### Candidacy identification

The first stage of the Candidacy Framework is identification of a health need.^21^

#### Self-recognition of SRH need

Self-recognition of an SRH need was reliant on several factors. Knowledge of SRH was the predominant barrier to seeking care and was crucial to the identification of candidacy. Individuals needed a certain level of health knowledge to understand the risk of STIs, and about pregnancy prevention and safe sex. Stigma and shame formed dominant themes, leading to a lack of discussion about SRH, which was a taboo subject among many communities:^^47,48,53,57–60^^

*‘I’d feel embarrassed cos then it won’t be a secret. If my parents were exposed to it as well, I would be more ashamed, then I wouldn’t be able to look at their face and talk to them face to face as I used* [to]*, cos I would know, that they know what I have now... especially if my mum was with me.’* (Asian female)^^48^^

This highlights the unique impact of sociocultural contexts on access.

Blame — both in terms of patients avoiding being blamed for their ‘risky’ behaviour or blaming others who may have caused their condition — prevented people from seeking diagnosis and care. There was also a fear of negative consequences, which made people not wish to seek a diagnosis, due to anticipated implications for themselves and others.^^48^^

#### Service recognition

Identifying accessible, acceptable, and effective health care relating to the SRH need was essential. For the individual to self-identify to a service, it had to be acceptable to their personal demographic and personal needs; confidentiality or perceived confidentiality was a vital aspect of this.^53,54^ The authors identified patients ’shopping around’ for GPs or practices to whom they felt more culturally aligned; this was more prominent in the HIV care group and the men-who-have-sex-with-men (MSM) groups. Many described putting substantial effort into ﬁnding a suitable GP:

*‘I researched my GP. I asked some people locally and went to four different surgeries and stayed with one, but I never get to see my named GP. I found out that one of them used to work in* [named London HIV clinic]*. I went to seek him and seek him out each time and he’s absolutely fantastic.’* (Male, MSM, comorbidity group)^^49^^

Although some participants preferred to access services via the relative anonymity and accessibility of general practice — where it is possible to attend with any health problem, compared with sexual health clinics — others feared the lack of confidentiality, especially if they were from smaller communities in rural areas.^^60^^

### Navigation of candidacy

The second stage of the Candidacy Framework is navigation of services, which refers to the process of seeking to assert candidacy.^21,23^ Navigation requires an understanding of what services are available and the ability to mobilise practical resources to access that service. For younger people, navigating sexual health services can be challenging — they may, for example, lack SRH service knowledge and practical resources, such as transport; as a result, they may rely on parents, family members, or peers to help them access care. Primary care seemed to have fewer barriers to access compared to secondary care (excluding emergency departments) in terms of practical resources needed, such as transport, ability to leave school or work, and the need for childcare. Barriers, such as missing work, travelling to sexual health clinics, and travel costs, were evident:^^57,61,62^^

*‘I was told to be at the clinic 90 minutes before they started testing at 1pm but I was there an hour early. They’d given out all the tickets at 11.30 am so I was too late. I then asked about the the other hospital clinic and was told about the six week wait.’* (Male, MSM, aged late 20s, tested negative for STI)^^57^^

SRH was a lower priority in individuals’ lives, especially in the context of deprivation. Other priorities, such as childcare, work, benefits, substance misuse, and probation, are more imminent. The idea of only accessing a GP when unwell was a predominant theme throughout the literature. Burns *et al* ^61^ concluded their discussion, *‘health is only a priority when one is unwell; otherwise, issues around immigration, housing, employment, and childcare take precedence’*. This highlights the challenges people have prioritising health, especially when they are well and have other demands or priorities.

### Permeability of services

Permeability is understood as the ease with which people can use services.^21^ The notion of permeability had clear resonance in the studies reviewed, with more than half of them mentioning barriers around getting an appointment in general practice.^42,43,45,46,49,51–54,56–62,64,72,73,78,80,82^ As stated in one study, drop-in services were a way of improving permeability:

*‘I feel it’s more suitable like youth based and I feel like they’ve got more time kind of thing if I need it. Because I know that GP clinics are busy and trying to get anappointment, you know it can be hard work.’* (Female, aged 18 years)^62^

This compares to the challenge of trying to get an appointment through telephone booking systems:

*‘Because it’s difﬁcult, it’s like a rat race here* [genito-urinary medicine clinic] *at 9 o’clock in the morning, and when I’ve just arrived at work, you know, spending all the time on the phone, it just really didn’t go down too well.’* (Female, 26 years old)^^62^^

One area in which issues around the permeability of services were very apparent was in the care of HIV, a condition that needs chronic disease management:

*‘Before, my GP was very good. If I have an issue and call for an appointment, if they have nothing for today, they will ﬁt me in the next day. With this one, they tell me to call back next day and each time I call, they tell me they are fully booked and to call back the next day.’* (Woman, African group)^^49^^

There was evidence that some general practices were aiding the permeability of services for patients with HIV by identifying them and ‘fitting them in’ for an appointment.^49^ Most people living with HIV will have a GP and a specialist; two articles described individuals as feeling as though they were ‘ping-pong’-ed between services.^^49,61^^

### Appearing and asserting at health services

This refers to the ability to self-present, communicate, and articulate the ‘need’ or issue to an HCP.^21^ In total, 20 studies referred to appearing or asserting oneself.^42,43,46–49,52,53,55,57,59–65,71,72,80^ The overarching theme through these studies was about discordance or disparity between patient and consulter. Gender and age of the HCP, compared with those of the patient, were highlighted as barriers to asserting candidacy in several studies:^47,50,52,57,60,63,65,72,80^

*‘I mean, if there is a similar age, it would be easy and open but if you see this GP being 60 years old, you would automatically think, OK, they are people who are conservative; I shouldn’t ask about sex. We should get younger GPs to help with the young people.’* (Male, aged 19 years)^^63^^

The term ‘stigma’ was used to encompass a range of different barriers, including shame, fear of judgement, fear of consequences, and embarrassment.^47,48,53,57–60^ One GP discussed how some topics may be more challenging to discuss:

*‘I think there are always difficulties (. . .) that the patient is willing to open up and address issues, fears, worries, needs, and also an erectile dysfunction or loss of libido (. . .).’* (GP)^^64^^

Another GP described how patients use different terms to try to explain their sexual activities:

*‘A classic quote one of the guys made was, he said he had “been out doing the traffic”* [’cruising’ in public places for a sexual partner or casual sex]. *He did not have to give an explanation of what he had been up to.’* (Male GP, high-frequency tester, small suburban practice)^^65^^

Language was a barrier to discussion. Two sub-themes emerged: first, how language and terminology might cause confusion and misunderstanding, especially the use of slang terms;^63^ second, that language could cause offence or make people feel excluded.^65^ In terms of potentially excluding people, a staff member in one study commented:

*‘It’s knowing how to deal with that* [misgendering someone] *‘cause some things that you are not so used to doing, you know and that’s where I think we need more support, so we know how to do these things, you know, otherwise it doesn’t come across too good.’* (Practice nurse)^^66^^

It was highlighted that patients would make a judgement about whether they could trust the HCP with whom they consulted:

*‘Trust..so I do say to them that it’s confidential, whatever you say won’t be shared with anybody else, but I think there’s still that bit of concern if it is an Asian Nurse or Muslim nurse. They will be like “shall I tell her, shall I not tell her… we don’t.. am not sexually active,” but they are sexually active because they are coming for a pill, the morning after pill, they are coming for some other things as well, so.. ’* (Female nurse)^^70^^

### Adjudication by HCP

This theme explores adjudication or judgements of worthiness by HCPs that influence subsequent management and intervention.^20^ Thirty-one studies contained themes related to adjudication, predominantly around LGBTQ+ issues, and stereotypes of age and ethnicity:^42,43,46,49–52,54–57,59–65,67–69,71–74,77–80,82,83^

*‘*[…] *because she (the woman) is usually a bit more sensible, I hope she says: “Hey, you, remember, do you have the condoms with you?”* (Female GP, aged 52 years)^^67^^

*‘Or prejudices or that it will be difficult for the doctor so that I don’t get good treatment, because he is so preoccupied with me being a lesbian, and that he then erects a barrier against me or something.’* (Woman, aged 28–59 years, self-identified as lesbian)^^59^^

*‘His attitude was that being gay was something that the Bible spoke against and perhaps I should reconsider my position.’* (Male, gay or bisexual)^^46^^

In some cases, the HCP described that they felt they could not maintain an objective opinion of someone who had different sexual practices to them.^58^ There were many examples of HCPs having homophobic or transphobic views, with doctors and nurses feeling more comfortable managing intimate issues for heterosexual patients.^42,58,68^ Some HCPs felt uncomfortable and ‘disagreed’ with being gay or transgender:

*‘I have relatively few* [barriers] *over heterosexual relationships; homosexual relationships I find a bit more difficult, prescribing Viagra for homosexual men I think is a bit dubious … I think it’s a slightly inappropriate use of resources really, but it’s probably my prejudices, I’m prepared to admit that … particularly if they are not in a stable relationship, I don’t see it’s appropriate.’* (Male GP, aged 50 years)^^69^^

In one study, there was a paradox in that the HCPs qualified such views with a statement that they still provided the same care.^45^ This lack of culturally congruent consultation and lack of confidence about how to deal with SRH in patients from the LGBTQ+ community created a barrier to providing effective care.^^58,60^^

### Offers and resistance to offer

This stage of candidacy focuses on the reasons why people might decline a service or an offer of treatment, and overlaps with previously mentioned themes, such as stigma and fear of judgement. This included fear of a positive result — for example, in relation to STI screening:

*‘Some people don’t like to know their results... they’d rather die... so it’s something like that, just scared of knowing what you’ve got.’* (Female, aged 17 years, Black Caribbean)^^45^^

It was also highlighted that some people might feel that a judgement was being made about the way they lived their lives:

‘Interviewer: *Why do you think people would be offended if someone brought it* [Chlamydia testing] *up with them?*

Respondent: *It’s just that you’re insinuating something about this person. You’re almost criticizing them, saying that they’re a certain type of person.’* (Female, aged late 20s, rural GP)^58^

### Operating conditions and local production of candidacy

#### General practice specific

By far the most predominant barrier for access was time restrictions of the general practice, whether perceived or actual.^48,54,59,61,63,70–77^ There was a perceived lack of time in consultations due to competing demands to cover all health topics. In the tight time constraints of general practice, it was highlighted that HCPs might avoid these taboo or sensitive SRH subjects:^^48,54,64^^

*‘Hypertensives for instance, gosh a lot of them cause impotence… I haven’t got anything to back this up with, but my feeling is that the sexual side-effects would be mostly neglected, cause it’s a sort of Pandora’s box isn’t it?… You don’t, sort of, want to open up all sorts of thing* [sic]*?’* (Female GP, aged 50–59 years)^^47^^

Both patients and HCPs showed reluctance in discussing SRH as it was not deemed to be part of general practice or, indeed, count as a valid medical condition. A study looking at older women with diabetes explored this; women felt happy to discuss diabetes, but awkward raising sexual topics.^56^ It was found that general practice offered the opportunity for a longitudinal relationship to form between patients and HCPs; this impacted how comfortable people felt disclosing personal topics.^54^ As well as time constraints being a barrier, loss of continuity in UK general practice challenged the ability to form cohesive longitudinal relationships with patients.^^78^^

With regards to patients with HIV in the Keogh study,^49^ this cohesive longitudinal relationship helped them to trust their GP and improved access.^^78^^

#### Health service culture

Sexual health was still seen as a taboo and a stigmatised area of health care, with secondary care services often having responsibility for its provision over primary care.^54,58^ This was deemed to be disadvantageous:

*‘It is not helpful to propagate the idea of ‘special infections’ that need to be treated in a ‘special place’… We need to demystify STIs among GPs, secondary care colleagues, and the public.’* (Consultant doctor)^^76^^

The NHS was, overall, a heteronormative sphere, in which heterosexuality was the default.^45,46^ In one study, a participant commented that:

*‘My sexuality has never been questioned. There’s been an assumption made that I’m heterosexual. I have this constant battle … and you just let it go on I suppose.’* (Woman, lesbian, aged 61 years)^^55^^

This default position made it challenging for minoritised sexual groups, members of whom had to negotiate discrimination.^43,54,58^ It was highlighted that such assumptions about sexuality meant that patients might be silenced or deterred from access, especially with regard to an SRH need:^^43,45,46,60^^

*‘And then he prescribed an ointment that I could apply, and then he prescribed something else that he said that my partner could apply on his genitals, and then I just had to say that ‘‘I am with a woman’’. Then he laughed and said ‘‘oh’’, and then he gave me two identical prescriptions.’* (Woman, aged 50–59 years, self-identified as lesbian)^59^

There was a variation in how different practices provided SRH services and health providers could often act reactively — for example, it was noted that, with pre-conceptual health interventions, they often only became engaged when the patient was already pregnant:

*‘... the reality is that I guess we’re more reactive only and “oh you’re pregnant” and we rely on somebody else, some other unknown persons, to advise that woman on pre-pregnancy care.’* (Practice nurse)^75^

There were examples of:

- proactive practice behaviour — people were actively sought for screening and offered services;^^48,69,73,76,77,82^^
- passive practice behaviour — people were offered no services and did not actively sought for screening;^55,73,82^ and
- reactive behaviour — practice or practitioner was happy to help if the patient raised an SRH issue.^65,73^

This fragmented and variable delivery of care led to inequality of provision.

## Discussion

### Summary

This review resulted in themes emerging in the context of improving universal access to SRH care via general practice by tackling issues such as confidence, language, stigma, taboo, knowledge, and literacy. The findings create a better understanding of how individuals and healthcare providers are impacted by societal, religious, and cultural belief systems when dealing with SRH. The impact of intersectionality was clear: disparities were layered, dependent on individual and group identities.^84^ Those at most risk of poor SRH outcomes were those experiencing combinations of discrimination and oppression.

### Strengths and limitations

A strength of this synthesis was the rigorous critical appraisal throughout, with adherence to PRISMA guidelines for the systematic review and use of the CASP checklist.^43,75^ The review formed part of a successful doctoral thesis that underwent peer review and critical appraisal throughout. Using a ‘best-fit’ approach allowed for the adaptation and development of alternative themes outside of the *a priori* framework.^31^

The Candidacy Framework offers a valuable and highly salient model for understanding access to SRH care in general practice. It provides a practical, theoretical framework to understand the complex interactional factors of access to SRH care. This review assimilated 42 studies with diverse topics, and participants included HCPs, service users, and members of the public. These diverse viewpoints help provide comprehensive perspectives of care journeys. The studies were from worldwide locations in high-income settings with various age groups.

This review explores the overarching themes associated with providing SRH care in general practice and, to the authors’ knowledge, this is the first systematic synthesis of multiple aspects of this type of health that have been explored. Rather than examining barriers in terms of specific subjects, such as chlamydia screening or LGBTQ+ consults, this project took a more holistic approach to try to understand the broader context of SRH care in general practice.

A limitation was the lack of diversity in ethnicity or inclusion of communities of socioeconomic deprivation in the published data. This is relevant as those with worse SRH outcomes are predominantly from minoritised ethnic groups.^84^ When ethnicity data were reported, a disproportionate number of young White people were included. This likely represents the challenges around recruitment and conducting qualitative research among minoritised ethnic groups.^^85,86^^

A further finding was the inconsistent use of ethnic groups, making the comparison between groups challenging. Some studies grouped people into ‘ethnic minority’, whereas others gave more detailed race demographics.^87^ In the US, studies by the National Institutes of Health must publish ethnicity, race, and sex of participants to improve equity and representation;^87^ there is no similar requirement in the UK and Europe.

There were apparent challenges representing the deprivation level of study participants. Some studies used employment, education, or income to proxy deprivation. This highlights the challenge of defining deprivation and the impact it has on good-quality evidence synthesis. There was also a bias towards the perceptions of HCPs, which might be explained by the challenges of recruiting patients and participants for SRH research.^88,89^

### Comparison with existing literature

This review highlights the importance of identifying needs in the candidacy journey. Crucial barriers were stigma, knowledge, and the ability to prioritise SRH. This was evident with STIs, which are often asymptomatic; as such, preventative measures and screening are essential.^70^ For this to happen, the individual must have prerequisite knowledge of infection to identify a need.^35,90^ Those at most risk of poor health outcomes will often find prioritising and mobilising the necessary assets needed to access care more challenging; this was well described in a study on medical homelessness for women transitioning from prison.^26^

The impacts of sociocultural context and religion were evident throughout the present review. A systematic review of the views and experiences of women from minoritised ethnic groups on LARC found similar findings, with external factors influencing choice.^91^ Acceptability or perceived adverse effects of contraception have also been found to impact uptake, with poverty and education playing a crucial role.^92^ The local production of candidacy or operating conditions were also relevant when looking at barriers to access in general practice. Critical barriers were time constraints, poor appointment availability, and time pressures. There was a sense that SRH topics needed more time and tact to discuss a sensitive subject area, and continuity of care was a key facilitator.^93,94^ Within the review, there were different countries with varied healthcare systems. These structural differences may impact provision of SRH care — as an example, the NHS is free at the point of contact, whereas some insurance-based systems may need initial financial outlay.

In the US, patients preferred physicians leading enquiries around SRH.^95^ Trust is an essential facet of communication, especially in this subject area. This links to the concepts of appearing and asserting candidacy. Paradoxically, HCPs prefer a more-passive consult, with patient-initiated discussion about sexual health, which concurs with current evidence.^27,95,96^ It seems common for providers to wait for patients to take the initiative, while patients wish for providers to initiate conversations concerning SRH.^27,29,95^ Present study findings explored this dynamic interplay, with each party in the consultation wanting the other to raise the SRH topic.

### Implications for research

The effort patients made to find a suitable GP in the studies identified in this review highlights the need for inclusive and open services with which people can identify. However, understanding access or use of healthcare services is complex and can be challenging to understand and research.^97^ It is imperative to provide services that are culturally congruent and take account of the variation in health literacy, knowledge, and acceptability of SRH. Previous studies had looked at healthcare access, in terms of utilisation, with an understanding that if a service is there, it will be used;^17,21,23,24^ Dixon-Woods *et al* offered the Candidacy Framework as a mechanism to better understand access for vulnerable groups. There is an opportunity to improve inequalities in health in the post-pandemic era; targeting the patchy and fragmented provision of SRH care is critical. There needs to be a focus on those for whom access is most challenging, particularly communities of socioeconomic deprivation.

This evidence synthesis formed the foundations of subsequent projects in which HCPs were interviewed in practices with high levels of deprivation to better understand barriers to accessing care; this was presented in a policy document^98^ to highlight the need for a focus on underserved populations. It further led to a participatory action research project exploring contraception access for those in racially minoritised groups.^99,100^

Researchers and grant providers have a responsibility to proactively engage with people from communities often excluded from research; examples include minoritised ethnic groups, people in areas of high deprivation, and LGBTQ+ communities. Without their input, they are invisible, their issues are not understood, research is not transferable, and it cannot be used to develop widespread intervention or service change.
