# Supplementary material for: Understanding access to sexual and reproductive health in general practice using an adapted Candidacy Framework: a systematic review and qualitative evidence synthesis
Source: Br J Gen Pract. 2025 Jun 17:BJGP.2024.0522. Online ahead of print. doi: 10.3399/BJGP.2024.0522 (PMC12199993; doi:10.3399/BJGP.2024.0522)
Supplement: Supplementary file 1 [file BJGP.2024.0522_suppl.pdf]

Supplementary Box S1 Inclusion and Exclusion criteria

| **Inclusion criteria** | **Exclusion criteria** |
|---|---|
| • General practice setting<br>• Published within the last 25 years<br>• Primary qualitative data, including mixed methods.<br>• High-income settings with universal access to general practice<br>• English language.<br>• Health topics include contraception, sexually transmitted infections, sexuality, and gender identity. | • Antenatal, pregnancy or postnatal, sexual dysfunction, erectile dysfunction, female genital mutilation, abortion.<br>• Community clinics not related to general practice (i.e. outreach clinics from secondary care)<br>• Sexual violence.<br>• Systematic or narrative reviews. |

## Supplementary Appendix S1 – Search terms

1. exp General Practice/

2. general practice.af.

3. General Practitioners/

4. General Practitioners.af.

5. Physicians, Family/

6. GP.af.

7. Primary Health Care/

8. primarycare.af.

9. primary care.af.

10. Family Practice/

11. family practice.af.

12. family medicine.af.

13. Physicians, Family/

14. family Physicians.af.

15. family practitioner.af.

16. primary healthcare.af.

17. primary health care.af.

18. Primary Health Care/ or Primary Care Nursing/ or Nurse Practitioners/

19. Primary Care Nursing.af.

20. Nurse Practitioners.af.

21. 1 or 2 or 3 or 4 or 5 or 6 or 7 or 8 or 9 or 10 or 11 or 12 or 13 or 14 or 15 or 16 or 17 or 18 or 19 or 20

22. Reproductive Health/

23. sexual health.af.

24. reproductive health.af.

25. pelvic infection/ or reproductive tract infections/ or exp sexually transmitted diseases/

26. Pregnancy, Unwanted/ or Pregnancy/

27. Contraception Behavior/ or Contraception/ or Contraception, Barrier/ or Contraception, Postcoital/ or contraception.mp.

28. Gender identity.mp. or Gender Identity/

29. exp sexual behavior/ or exp sexuality/ or exp unsafe sex/

30. Abortion, Induced/ or Family Planning Services/

31. transgender.mp. or Transgender Persons/

32. sex workers/ or exp sexual minorities/

33. Contraception, Postcoital/

34. 22 or 23 or 24 or 25 or 26 or 27 or 28 or 29 or 30 or 31 or 32 or 33

35. 21 and 34

36. Health Services Accessibility/ or Professional-Patient Relations/

37. "Attitude of Health Personnel"/

38. barrier.ab,ti.

39. facilitator.ab,ti.

40. "barrier*".ab,ti.

41. "facilitator*".ab,ti.

42. "delivery of health care, integrated"/ or health services accessibility/ or health equity/

43. 36 or 37 or

Table S1 Summary of included studies

| Author. Year. | Location of research | Focus of study | Journal | Data collection method | Participants | Title | Ethnicity data included |
|---|---|---|---|---|---|---|---|
| Adakpa 2024 (70) | England | Young people | Journal of Family Medicine and Primary Care | Interviews | Practice staff (N= 7) | Engaging young people with sexual health services in general practice surgeries – A qualitative study of health care professionals | N/A |
| Balfe 2009(57) | Ireland | Young people | BMC Public Health | Interview | Service users (N = 30) | What prompts young adults in Ireland to attend health services for STI testing? | No |
| Balfe 2010(58) | Ireland | Young women | BMC Public Health | Interview | Service users (N = 35) | Young women's decisions to accept chlamydia screening: influences of stigma and doctor- patient interactions | No |
| Beagan 2015(71) | Canada | LGBTQ | Canadian medical education journal | Interview | GP (N = 24) | Family physician perceptions of working with LGBTQ patients: physician training needs. | N/A |
| Bjorkman 2007(59) | Norway | Same-sex-attracted women | Scandinavian Journal of Primary Health Care | Focus group | Public (N = 6) | Being lesbian – does the doctor need to know? | No |
| Burns 2007(61) | England | HIV | AIDS Care - Psychological and Socio-Medical Aspects of AIDS/HIV | Interview | Experts (N = 11) | Why the(y) wait? Key informant understandings of factors contributing to late presentation and poor utilisation of HIV health and social care services by African migrants in Britain | N/A |
| Cant 2002(78) | England | MSM | Primary Health Care Research | Interview | Public (N = 17) | An exploration of the views of gay and bisexual men in one London borough of both their primary care needs and the practice of primary care practitioners | Brief |

| | | | | | | | |
|---|---|---|---|---|---|---|---|
| | | | and Development | | | | |
| Cant 2006(46) | England | MSM | Health and Social Care in the Community | Interview | Public and managers (N = 50) | Exploring the implications for health professionals of men coming out as gay in healthcare settings | Extensive |
| Carroll 2024 (66) | New Zealand | LGBTQ | Family Practice | Focus group | Practice staff (n= 25) | “It was a very awkward consultation because I didn’t know”—general practice staff experiences and challenges in providing healthcare to gender and sexual minority youth in Aotearoa New Zealand | N/A |
| Collyer 2018(72) | Australia | Young men | Australian Journal of General Practice | Interview | GP (N = 17) | General practitioners’ perspectives on promoting sexual health to young men | N/A |
| Dixon-Woods 2001(54) | England | Women | Sexually transmitted infections | Interview | Sexual health service users (N = 37) | Choosing and using services for sexual health: a qualitative study of women's views. | Brief |
| Ejegi-Memeh 2021(56) | England | Women with diabetes | Journal of advanced nursing | Interpretative phenomenological | Member of the public (N = 10) | Sexual health discussions between healthcare professionals and midlife-older women living with Type 2 diabetes: An interpretative phenomenological study | Brief |
| Ewert 2016(43) | Australia | Young men | Sexual Health | Interview | Students (N = 28) | 'Most young men think you have to be naked in front of the GP': A qualitative study of male university students' views on barriers to sexual health | No |
| Fairhurst 2005(73) | Scotland | Emergency contraception | Family Practice | Interview | Mixed healthcare professionals (N = 11) | “Not that sort of practice”: the views and behaviour of primary care practitioners in a study of advance provision of emergency contraception | N/A |
| Freeman 2009(74) | England | Chlamydia | BMC Public Health | Focus group | Staff in general practice (N = 25) | Promoting chlamydia screening with posters and leaflets in general practice - a qualitative study | N/A |

| | | | | | | | |
|---|---|---|---|---|---|---|---|
| Gott 2004(47) | England | Sexual health | Family Practice | Interview | GP and practice nurse (N = 57) | “Opening a can of worms”: GP and practice nurse barriers to talking about sexual health in primary care | N/A |
| Gott 2003(52) | England | Older adults | Family Practice | Interview | GP and patients (N = 45) | Barriers to seeking treatment for sexual problems in primary care: a qualitative study with older people | No |
| Hall 2024 (75) | Great Britain | Pregnancy intention | BJGP Open | Online Interviews | GP, health visitors, practice nurse, community sexual and reproductive health nurses and doctors. (n = 12) | How, when and who should ask about pregnancy intentions in primary care? A qualitative study of primary health care professionals’ preferences | N/A |
| Heritage 2008(48) | England | Young people | Reproductive Health | Focus group | School students and GP patients (N = 18) | A study of young peoples' attitudes to opportunistic Chlamydia testing in UK general practice | Brief |
| Hinchliff 2004(80) | England | Gender-barriers | European Journal of General practice | Interview | GP (N = 22) | GPs' perceptions of the gender-related barriers to discussing sexual health in consultations A qualitative study | N/A |
| Hinchliff 2005(69) | England | LGBTQ | Health and Social Care in the Community | Interview | GP (N = 22) | 'I daresay I might find it embarrassing': General practitioners' perspectives on discussing sexual health issues with lesbian and gay patients | N/A |
| Hocking 2008(68) | Australia | Chlamydia | BMC Public Health | Interview and questionnaire | GP (N = 21) | What needs to change to increase chlamydia screening in general practice in Australia? The views of general practitioners. | N/A |

| | | | | | | | |
|---|---|---|---|---|---|---|---|
| Hogan 2010(60) | England | Young people | BMC Public Health | Interview | GP patients (N = 36) | '...they should be offering it': a qualitative study to investigate young peoples' attitudes towards chlamydia screening in GP surgeries | No |
| Joore 2017(80) | Netherlands | HIV | International Journal of STD & AIDS | Interview and focus group | GP (N = 90) | General practitioners' barriers and facilitators towards new provider-initiated HIV testing strategies: a qualitative study | N/A |
| Keogh 2016(49) | England | HIV | Primary Health Care Research and Development | Focus group | Sexual health service users and public (N = 51) | Learning from the experiences of people with HIV using general practitioner services in London: A qualitative study | Extensive |
| Latreille 2014(63) | Australia | Young men | Australian family physician | Interview | Students (N = 31) | Finding a segue into sex: young men's views on discussing sexual health with a GP. | No |
| Llewellyn 2012(53) | England | STI testing | Sexually transmitted infections | Focus group | Service users (N = 65) | Understanding patient choices for attending sexually transmitted infection testing services: a qualitative study. | Extensive |
| Lorch 2015(50) | Australia | Chlamydia | BMC Family Practice | Interview | Practice nurses (N = 23) | Practice nurse chlamydia testing in Australian general practice: a qualitative study of benefits, barriers and facilitators | N/A |
| Lunniss 2016(51) | Scotland | Contraception | Journal of Family Planning and Reproductive Health Care | Interview | GP (N = 13) | Views of general practitioners on providing contraceptive advice and long-acting reversible contraception at the 6-week postnatal visit: A qualitative study | N/A |
| Ma 2005(76) | England | Chlamydia | Journal of Family Planning and Reproductive Health Care | Interview | Mixed healthcare professionals (N = 12) | Chlamydia screening in general practice: views of professionals on the key elements of a successful programme | N/A |

| | | | | | | | |
|---|---|---|---|---|---|---|---|
| Malta 2020(44) | Australia | Older adults | Australian journal of ageing | Interview | Members of public (N = 21) | 'That might be a bit sexy for somebody your age': Older adult sexual health conversations in primary care | No |
| Meurer 2023 (67) | Germany | STI | European Journal of General Practice | Interviews | GP (N = 19) | Women's sexually transmitted infections in primary care: General practitioners' challenges and strategies – A qualitative study in Germany | N/A |
| Mikulak 2021(81) | UK | Young trans people | British Journal of General Practice | Interviews | Health professionals (N = 20) | Health professionals' identified barriers to trans healthcare: a qualitative interview study | N/A |
| McNair 2015(55) | Australia | Same-sex-attracted women | Australian family physician | Interview | GPs and public (N = 60) | Disclosure for same-sex attracted women enhancing the quality of the patient-doctor relationship in general practice. | Brief |
| McNulty 2004(82) | England | Chlamydia | British Journal of General Practice | Focus group | Staff in general practice (12 focus groups) | Barriers to opportunistic chlamydia testing in primary care | N/A |
| Newman 2013(77) | Australia | HIV | Sexual Health | Interview | GPs and experts (N = 71) | Engaging non-HIV specialist general practitioners with new priorities in HIV prevention and treatment: qualitative insights from those working in the field | N/A |
| Normansell 2016(45) | England | Sexual health | Health Expectations | Interview | Students (N = 17) | Exploring access and attitudes to regular sexually transmitted infection screening: The views of young, multi-ethnic, inner-city, female students | Extensive |
| Pavlin 2008(83) | Australia | Chlamydia | BMC Infectious Diseases | Interview | GP patients (N = 24) | Take the sex out of STI screening! Views of young women on implementing chlamydia screening in General Practice | No |
| Sutcliffe 2011(62) | England | Sexual health | Sexually transmitted infections | Interview | Service users (N = 49) | Comparing expectations and experiences of care for sexually transmitted infections in general practice: a qualitative study. | Extensive |
| Tuomainen 2013(42) | England | Preconception health | BMJ Open | Interview and focus group | Public (N = 41) | Opportunities and challenges for enhancing preconception health in primary care: qualitative study with women from ethnically diverse communities | Extensive |

| | | | | | | | |
|---|---|---|---|---|---|---|---|
| Woodbridge 2015(65) | New Zealand | HIV | Journal of primary health care | Interview | GP (N = 17) | 'He said he had been out doing the traffic': general practitioner perceptions of sexually transmitted infection and HIV testing strategies for men. | N/A |
| Zannoni 2021 (64) | Germany | Sexual Issues | Journal of Primary Care and Community Health | Interview | GP (N = 16) | Addressing Sexual Problems in German Primary Care: A Qualitative Study | N/A |
